# Supplementary material for: Insulin Elevates ID2 Expression in Trophoblasts and Aggravates Preeclampsia in Obese ASB4-Null Mice
Source: Int J Mol Sci. 2023 Jan 21;24(3):2149. doi: 10.3390/ijms24032149 (PMC9917068; doi:10.3390/ijms24032149)
Supplement: Supplementary file 1 [file ijms-24-02149-s001.zip › 2.8 ijms-2104882-ONLINE SUPPLEMENT-10042022-Final.pdf]

ONLINE SUPPLEMENT

Insulin Elevates ID2 Expression in Trophoblasts and Aggravates Preeclampsia in Obese ASB4-Null Mice

Yukako Kayashima[1], W.H. Davin Townley-Tilson[1], Neeta L. Vora[2], Kim Boggess[2], Jonathon W. Homeister[1], Nobuyo Maeda-Smithies , Feng Li[1]*

[1]Dept of Pathology and Laboratory Medicine, The University of North Carolina, Chapel Hill, NC 27599, USA.

[2]Department of Obstetrics and Gynecology, Division of Maternal Fetal Medicine, The University of North Carolina, Chapel Hill, NC 27599, USA.

*Correspondence to: Feng Li, Dept of Pathology and Laboratory Medicine, The University of North Carolina, Chapel Hill, NC 27599, USA.

Phone: 919-966-6915. Fax: 919-966-8800.

E-mail: Feng_Li@med.unc.edu

Materials and Methods

Plasma biochemical measurements: At 18.5 dpc, blood was collected from mice after 4 h fasting. Plasma sFLT1, VEGF, Leptin, and insulin were measured using ELISA kits. (#MVR100, #MMV00, R&D Systems, Inc.; #KMC2281, Invitrogen; #90060, Crystal Chem) (12, 48). Plasma glucose, cholesterol and triglycerides were measured by a respective kit (Wako Chemicals, Richmond, VA) (48).

Immunofluorescence (IF): IF was performed as described in Waldo, et al. (49), on placental tissue sections at the indicated embryonic day. Briefly, tissue was harvested and fixed in 4% paraformaldehyde overnight with subsequent cryoprotection in 30% sucrose. Samples were embedded in OTC Compound (Sakura Finetek, Torrance, CA, USA) and sectioned into 6-µm thick slices by the UNC Histology Research Core Facility. Tissues were stained overnight with ID2 antibody (MA5-32891, Invitrogen, Rockford, IL) per manufacturer protocols, and visualized with Alexa Fluor 488 (Invitrogen, Eugene, OR) for one hour at room temperature in the dark. Negative controls were prepared as above in the absence of primary antibody. Tissues were imaged on an Olympus BX61 upright fluorescent microscope with an exposure time of 200 ms, and zero gain. Image montages were batch processed in FIJI (http://rsbweb.nih.gov/ij/) for quantification and intensity measurements

Western blot (WB): Snap frozen tissues or fresh cells were lysed in a radioimmunoprecipitation assay (RIPA) buffer and the protein concentration was determined by BCA protein assay kit (Thermo scientific, IL). Total protein of 20 µg/lane was subjected to 10%–20% SDS-PAGE, then electrotransferred onto PVDF membranes. The chemiluminescent intensities of the targeted protein bands were captured using the ODYSSEY® FC system and evaluated using Image Studio Software (LI-COR Biosciences, Lincoln, NE). Individual protein level was quantified by normalizing its intensity to the β-actin in the same sample and expressed

relative to the mean levels of the respective control group (set as one). The antibodies used in the study include: ID2 (MA5-32891, Invitrogen) and β-actin (#5125; Cell Signaling).

Morphological examination: Kidney tissues and adipose tissues were fixed with 4% paraformaldehyde, paraffin sectioned (5 μm), and stained with Periodic Acid-Schiff (PAS), or with Masson's Trichrome. Adipose tissues were stained with hematoxylin and eosin (H&E). The total number of glomeruli was counted in a section with PAS staining (approximately 80 to 110 glomeruli/section) and the number impaired glomeruli (defined by blocked capillary lumen) was also counted in the same section. The number of impaired glomeruli was expressed as a percentage of the total number of glomeruli examined. One section per mouse (3 mice from each group) was examined. Histological analysis was done by one well-trained scientist who was blinded to the experimental groups. Pregnant females and embryos/fetuses were examined at different time points: 12.5, 13.5 and 18.5 dpc. Fixed placentas (4% paraformaldehyde) were sectioned (5 μm) and stained with hematoxylin and eosin (H&E)

Quantitative RT-PCR: Total RNA from tissues or cells was extracted using Trizol (Life Technologies, St. Paul, MN) following the manufacturer's instruction. NanoDrop spectrophotometer method and gel electrophoresis was used to check quantity and quality of RNA. mRNA was quantified with TaqMan real-time quantitative RT-PCR (7500 real time PCR system, Applied Biosystems, Foster City, CA) by using one-step RT-PCR Kit (Bio Rad, Hercules, CA) with *18s* as reference genes in each reaction for mouse samples and *GAPDH* for human cells. (18, 43). Sequences of primes and probes are shown in Table S3.

Table S1. The effects of diet and genotype on pregnant WT and *Asb4*$^{-/-}$ mice

Parameters	NC-WT	HFD-WT	NC-*Asb4*$^{-/-}$	HFD-*Asb4*$^{-/-}$	p (ANOVA)		
					G	D	G x D
Body weight (g)	28.8 ± 0.5	29.2 ± 0.3	32.3 ± 0.9[a]	30.5 ± 0.8	<0.01	0.23	0.11
Visceral fat (g)	0.25 ± 0.02	0.40 ± 0.09	0.42± 0.04	0.80 ± 0.10[b]	<0.01	<0.01	0.08
Subcutaneous fat (g)	0.26 ± 0.02	0.30 ± 0.02	0.35 ± 0.03	0.42 ± 0.03[a]	<0.01.	0.04	0.71
Brown fat (g)	0.063 ± 0.006	0.062 ± 0.002	0.099 ± 0.005[a]	0.105 ± 0.008[a]	<0.01	0.70	0.61
p-cholesterol (mg/dL)	72.4 ± 5.1	100.0 ± 5.6	75.2 ± 5.2	123.5 ± 12.0[c]	0.13	<0.01	0.23
p-triglyceride (mg/dL)	94.0 ± 11.3	61.5 ± 3.9	98.0 ± 16.7	78.1 ± 10.2	0.37	0.055	0.58
p-glucose (mg/dL)	135.7 ± 9.9	137.9 ± 6.8	123.2 ± 5.6	120.3 ± 8.5	0.07	0.97	0.75
p-insulin (ng/ml)	0.18 ± 0.06	0.29 ± 0.06	0.34 ± 0.04	0.98 ± 0.19[b]	<0.01	0.01	0.04
Sample size (n)	8	8	8	10			

WT and *Asb4*$^{-/-}$ female mice were randomly enrolled into a normal chow (NC) or a high fat diet (HFD) group at time of weaning. Four weeks later, they were mated with respective males and dams were sacrificed at 18.5 dpc after 4 h fasting. Tissues were collected and weighted. Two way-ANOVA was used with genotype (G) and diet (D) as two factors, GxD: interaction between genotype and diet. p: plasma. [a]: p<0.05 vs. WT groups; [b]: p<0.05 vs. other groups; [c]: p<0.05 vs. NC groups. The *Asb4*$^{-/-}$ dams on HFD had lower BW than *Asb4*$^{-/-}$ dams on NC, which was because they lost more fetuses than *Asb4*$^{-/-}$ dams on NC.

Table S2. The effects of diet and pregnancy on body fat and plasma biochemicals of *Asb4*$^{-/-}$ female mice

Parameters	NC-NP	HFD-NP	NC-pregnant	HFD-pregnant	p (ANOVA)		
					P	D	P x D
Body weight (g)	21.9 ± 0.73	22.6 ± 1.10	32.3 ± 0.90[a]	30.5 ± 0.80[a]	<0.01	0.39	0.10
Visceral fat (g)	0.71 ± 0.13	1.25 ± 0.22[b]	0.42 ± 0.04	0.80 ± 0.10	<0.01	<0.01	0.57
Subcutaneous fat (g)	0.21 ± 0.03	0.34 ± 0.07	0.35 ± 0.03[a]	0.43 ± 0.03[a]	<0.01	0.01	0.60
Brown fat (g)	0.066 ± 0.009	0.091 ± 0.007	0.099 ± 0.005	0.105 ± 0.008[c]	0.019	0.08	0.30
p-cholesterol (mg/dL)	141.4 ± 11.7[b]	165.5 ± 13.8[b]	75.2 ± 5.2	123.5 ± 12.0[b]	<0.01	<0.01	0.30
p-triglyceride (mg/dL)	54.0 ± 4.8	60.6 ± 9.6	98.0 ± 16.7	78.1 ± 10.2	0.02	0.59	0.29
p-glucose (mg/dL)	143.9 ± 10.7	172.0 ± 39.6	123.2 ± 5.6	120.3 ± 8.5	0.03	0.42	0.32
p-insulin (ng/ml)	0.30 ± 0.08	0.71 ± 0.10	0.34 ± 0.04	0.98 ± 0.19[d]	0.33	<0.01	0.49
Sample size (n)	6	4	8	10			

Asb4$^{-/-}$ littermates of female mice were randomly enrolled into a normal chow (NC) or a high fat diet (HFD) group at time of weaning. Four wks later, some mice were mated with ASB4-null males and dams were sacrificed at 18.5 dpc after 4 hr fasting. Non-pregnant females were also sacrificed at the same time point. Tissues were collected and weighted. NC-NP: ASB4-null non-pregnant (NP) female mice with normal chow diet (NC). HFD-NP: ASB4-null non-pregnant (NP) female mice with a high fat diet (HFD). NC-pregnant: ASB4-null pregnant female mice with normal chow diet (NC). HFD-pregnant: ASB4-null pregnant female mice with a high fat diet (HFD).Two way-ANOVA was used with pregnancy (P) and diet (D) as two factors, PxD: interaction between pregnancy and diet. p: plasma [a]: p<0.05 vs. NP groups. [b]: p<0.05 vs. NC-pregnant group. [c]: p<0.05 vs. NC-NP group. [d]: p<0.05 vs. NC groups.

Table S3. Primers and probes for qRT-PCR

Gene	Type	Sequence (5'-3')
m-Vegf	*Forward*	*CGG TTT AAA TCC TGG AGC GT*
	Reverse	ACG TCT GCG GAT CTT GGA CA
	Probe	FAM-CT GTG AGC CTT GTT CAG AGC GGA G- TAMRA
h-ID2	Hs04187239_m1 (Applied Biosystems)	
m-Id2	Mm00711781-m1 (Applied Biosystems)	
h-ASB4	Hs00211557-m1 (Applied Biosystems)	
h-ASB4	Forward	CAC ATC AGA TGG AAC ACA AAG T
	Reverse	CAC ACA GTA AAG AGA GAG TGG T
	Probe	FAM-AG CTA TCC CCG ATG ATG ACT TGG AG—TAMRA
18s	Forward	AGA AAC GGC TAC CAC ATC CA
	Reverse	CTC GAA AGA GTC CTG TAT TGT
	Probe	FAM-AG G CAG CAG GCG CGC AAA TTA C—TAMRA
h-GAPDH	Forward	GAA GGT GAA GGT CGG AGT C
	Reverse	GAA GAT GGT GAT GGG ATT TC
	Probe	FAM-CA AGC TTC CCG TTC TCA GCC- TAMRA

m:mouse, *h*:human

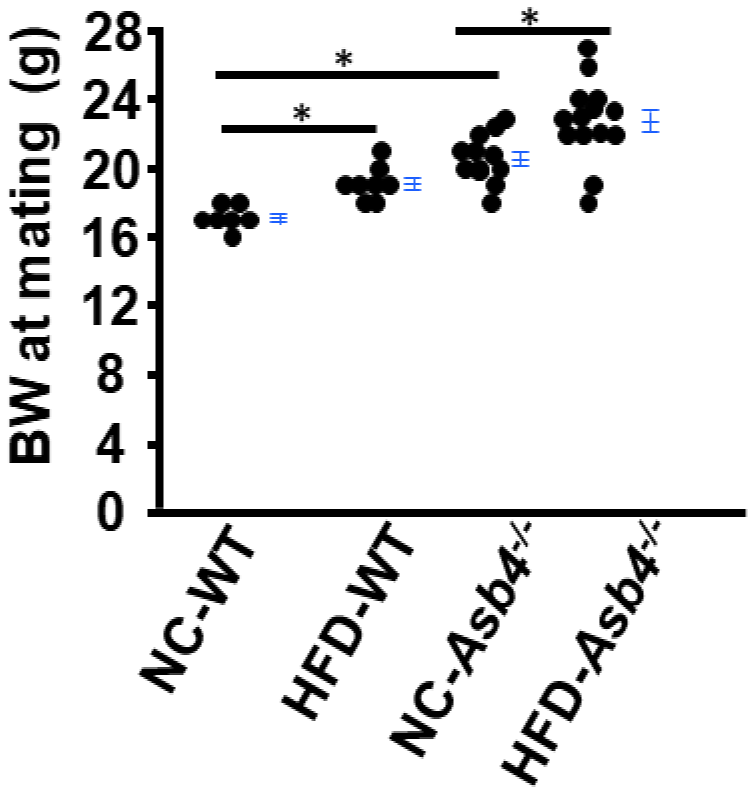

Figure S1. The body weight of four groups of female mice at the time of mating.

WT and *Asb4*$^{-/-}$ female mice were enrolled into normal chow (NC) or high fat diet (HFD) at the time of weaning (~21-23 day old). The body weight of these mice was measured 5 weeks after different diets. *p<0.05. n=7-15.

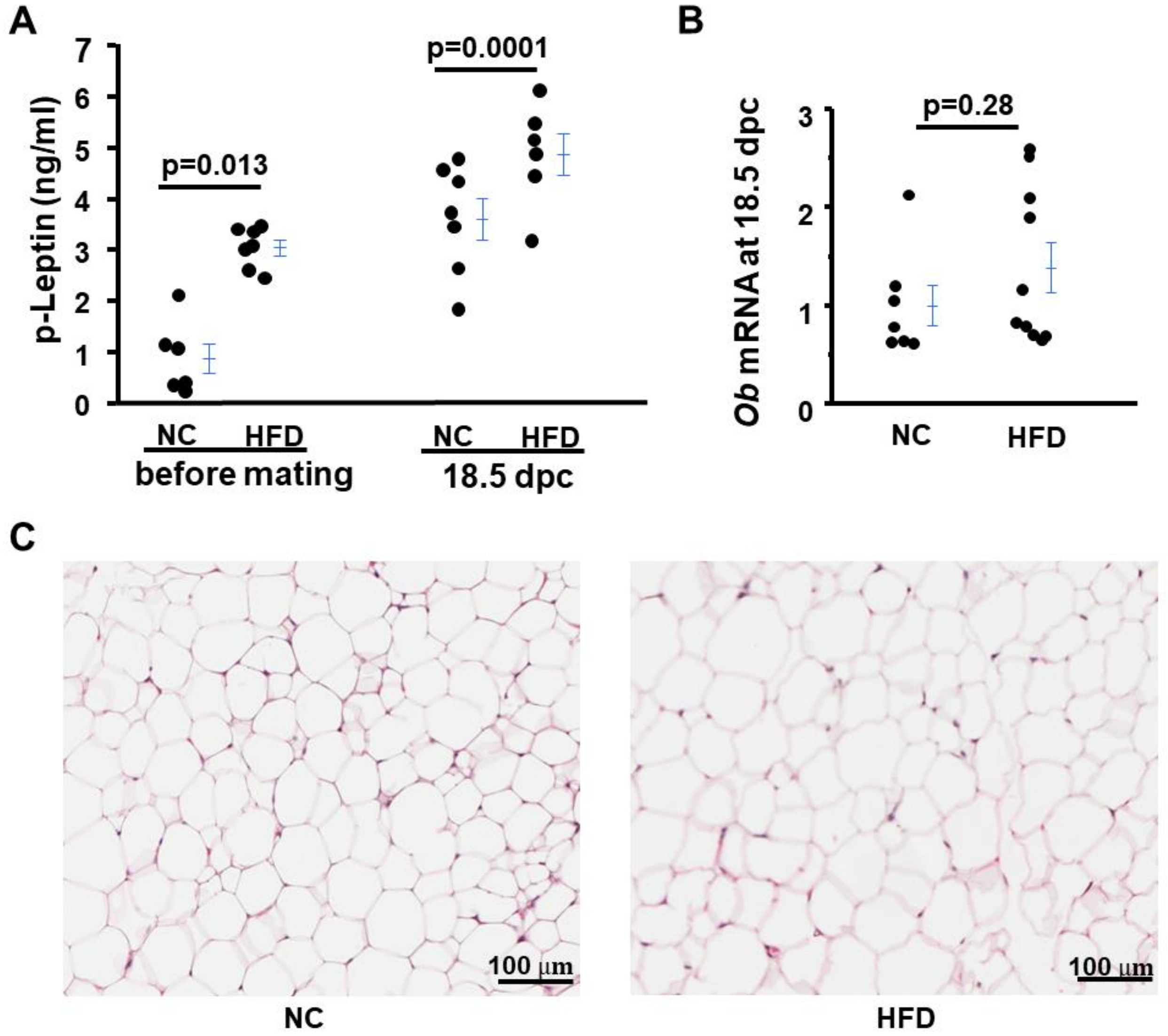

Figure S2. A high fat diet (HFD) increases plasma levels of leptin in *Asb4*$^{-/-}$ female mice. A. plasma levels of leptin at different stages of pregnancy. n=6-7. B. mRNA levels of *Lep* in gonadal adipose tissues at 18.5 dpc. n=7-9 C. Gonadal adipose tissue from NC- HFD- *Asb4*$^{-/-}$ dams at 18.5 dpc. H&E staining.

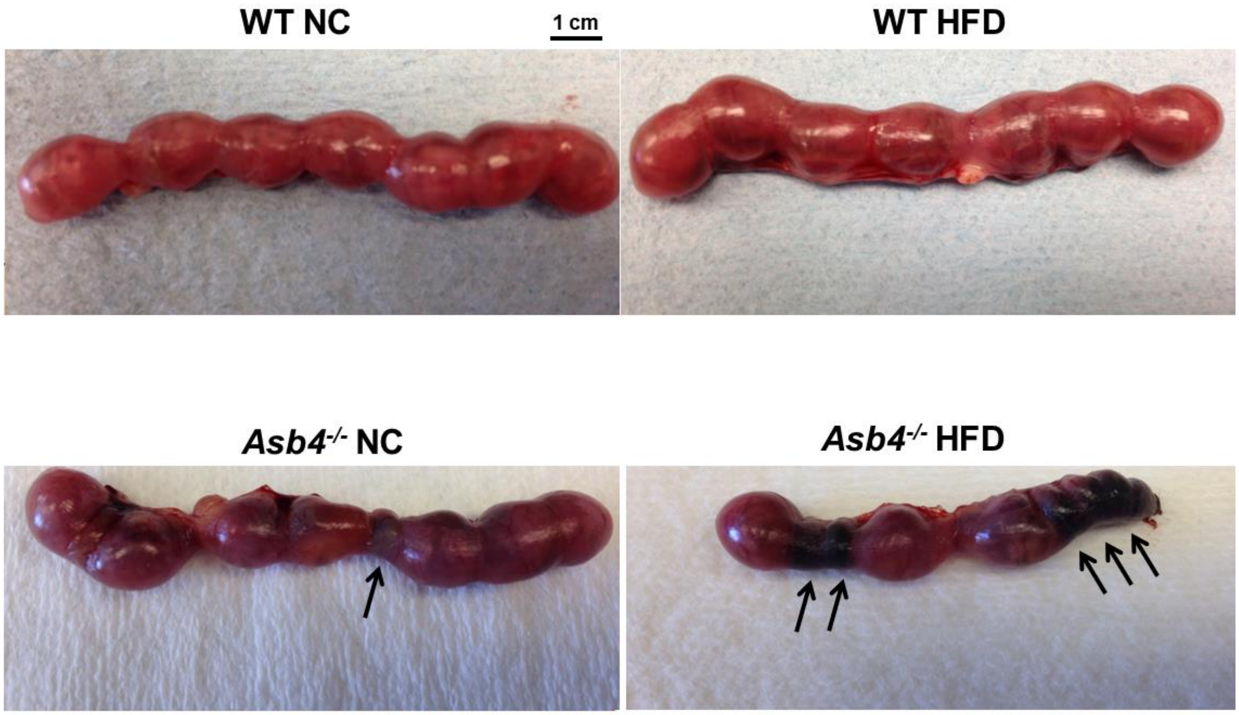

Figure S3. ***Asb4***$^{-/-}$ **dams fed a high fat diet (HFD) have more reabsorbed conceptuses than other groups of dams.** At 13.5 dpc, pregnant mice were euthanized and uteri were isolated and total conceptuses were counted. Arrows: reabsorbed conceptuses. NC: normal chow.

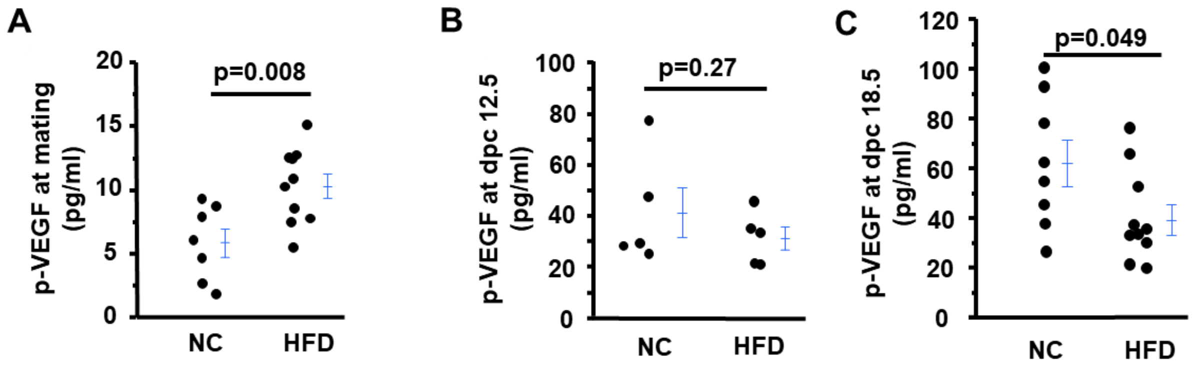

Figure S4. The levels of plasma VEGF at different stage of pregnancy in *Asb4*$^{-/-}$ female mice. n=5-9.

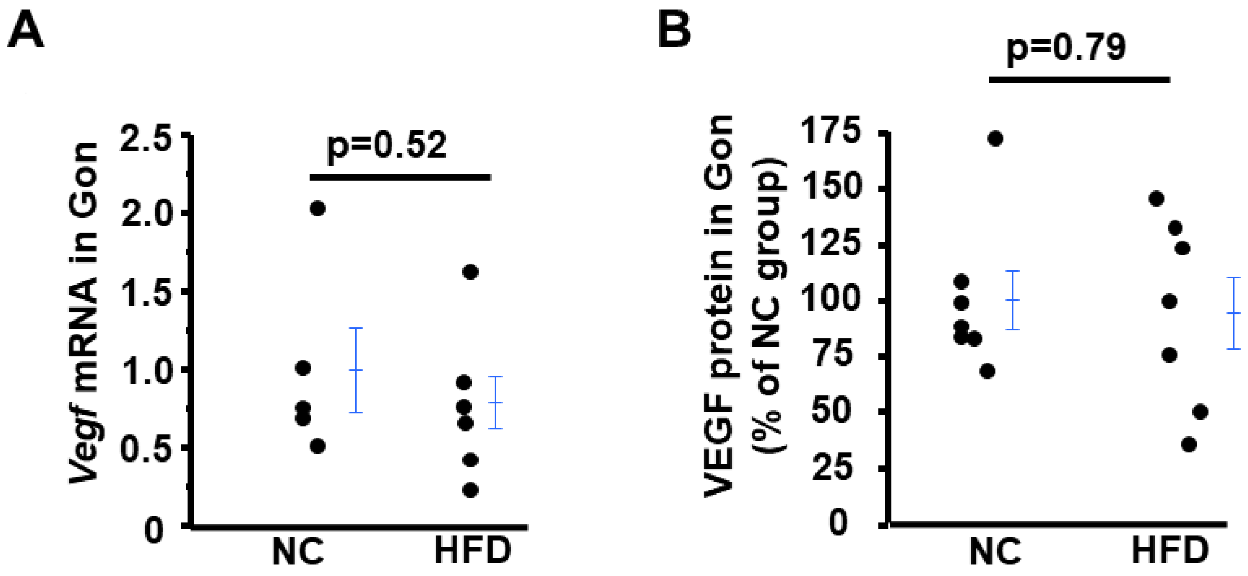

Figure S5. **The expressions of vascular endothelial growth factor (VEGF) in gonadal adipose tissues are not different between *Asb4*$^{-/-}$ dams fed normal chow (NC) and *Asb4*$^{-/-}$ dams fed a high fat diet (HFD).** Adipose tissues were collected at 18.5 day post coitus after *Asb4*$^{-/-}$ female pregnant mice were euthanized. A. mRNA levels of *Vegf*. B. Protein levels of VEGF. n=5-7.

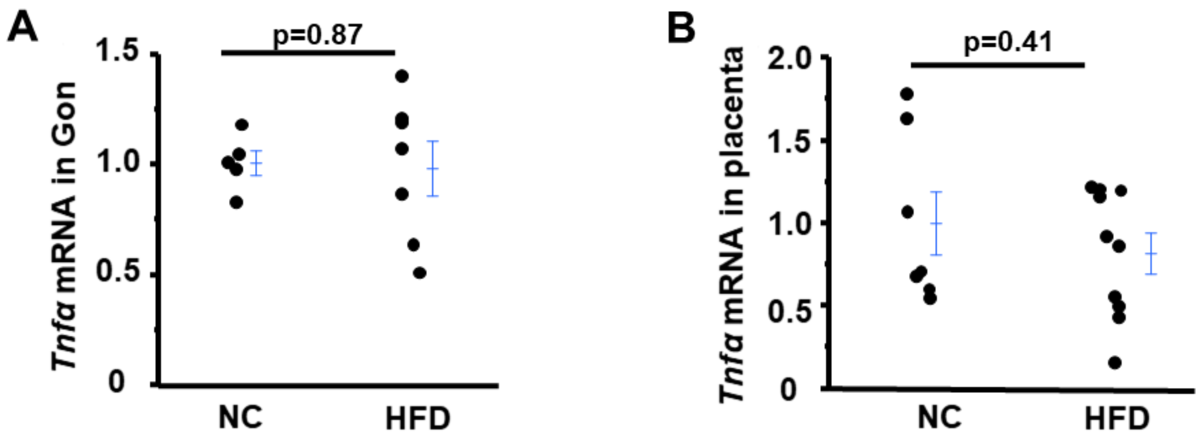

Figure S6. **The expressions of TNFα in gonadal adipose tissues and placentas are not different between *Asb4*$^{-/-}$ dams fed normal chow (NC) and*Asb4*$^{-/-}$ dams fed a high fat diet (HFD).** Gonadal Adipose tissues (Gon) and placentas were collected at 16.5- and 18.5-day post coitus respectively after *Asb4*$^{-/-}$ female pregnant mice were euthanized. A. mRNA levels of *Tnfa* in adipose tissues B. mRNA levels of *Tnfa* in placentas. n=5-9.

A

group	Ct(*GAPDH*)	Ct(*ASB4*)
human placental sample 1	27.2383	36.4585
human placental sample 2	26.3648	36.692
human placental sample 3	25.6437	34.9551
human placental sample 4	25.929	35.3651
human placental sample 5	24.3416	35.4755
human placental sample 6	24.1164	35.6119
HTR8 cell sample 1	22.2807	UD
HTR8 cell sample 2	22.4224	UD
HTR8 cell sample 3	21.412	UD
HTR8 cell sample 4	21.311	UD
HTR8 cell sample 5	22.036	UD
HTR8 cell sample 6	22.354	UD

B

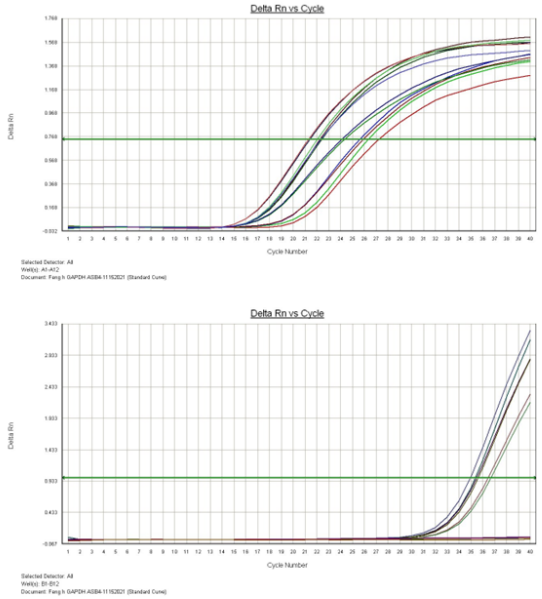

Figure S7. HTR8 trophoblast cells do not express *ASB4* determined by qRT-PCR. Six RNA samples of human placenta (kindly provide by Dr.Neeta Vora, co-author) and six RNA samples of HTR8 cells were subjected for qRT-PCR analysis using the primers and probe from Applied Biosystems (Hs00211557-m1). A. Ct of each sample. Note: all samples had housekeeping gene (GAPDH) expression. Human placental samples expressed *ASB4*, however, none of HTR8 samples expressed *ASB4*. UD: undetermined. B. Amplot of two genes. Upper panel: *GAPDH*, lower panel: *ASB4*.

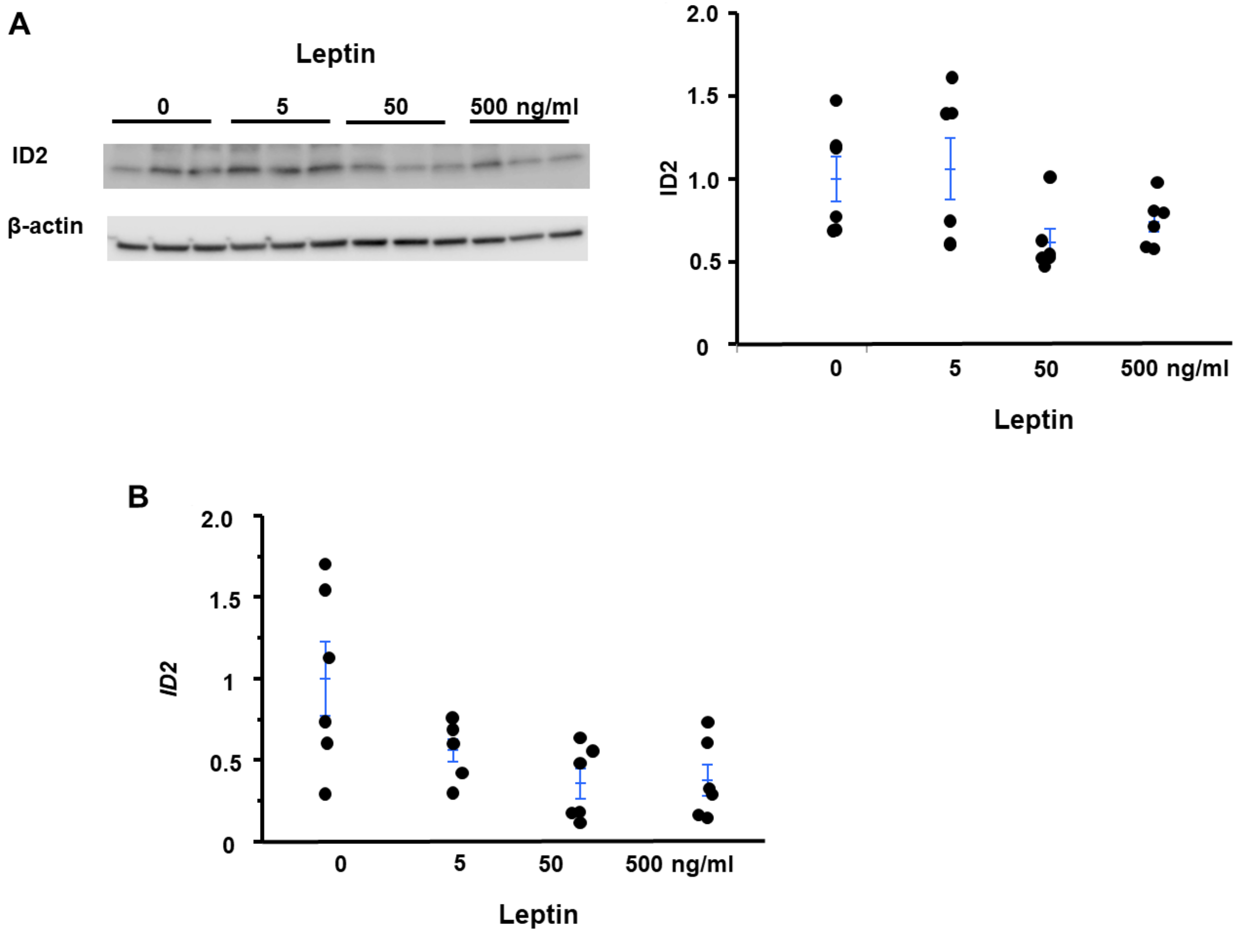

Figure S8. Leptin does not increase ID2 expression in HTR8 trophoblast cells. A. Western blot and densitometric quantitation of ID2. B. mRNA levels of *ID2*. n=6
